# Low oxygen levels caused by *Noctiluca scintillans* bloom kills corals in Gulf of Mannar, India

**DOI:** 10.1038/s41598-020-79152-x

**Published:** 2020-12-17

**Authors:** K. Diraviya Raj, G. Mathews, David O. Obura, R. L. Laju, M. Selva Bharath, P. Dinesh Kumar, A. Arasamuthu, T. K. Ashok Kumar, J. K. Patterson Edward

**Affiliations:** 1 Suganthi Devadason Marine Research Institute, 44-Beach Road, Tuticorin, 628001 Tamil Nadu India; 2 CORDIO East Africa, P.O. Box 10135, Mombasa, 80101 Kenya; 3 Gulf of Mannar Marine National Park, Ramanathapuram, 623 503 India

**Keywords:** Ecology, Climate sciences

## Abstract

Coral reefs around the world are undergoing severe decline in the past few decades. Mass coral mortalities have predominantly been reported to be caused by coral bleaching or disease outbreaks. Temporary hypoxic conditions caused by algal blooms can trigger mass coral mortalities though are reported rarely. In this study in Gulf of Mannar (GoM), southeast India, we report a significant coral mortality caused by a bloom of the ciguatoxic dinoflagellate *Noctiluca scintillans* during September–October 2019. Dissolved oxygen levels declined below 2 mg l^−1^ during the bloom causing temporary hypoxia and mortality (up to 71.23%) in the fast growing coral genera *Acropora*, *Montipora* and *Pocillopora*. Due to global climate change, more frequent and larger algal blooms are likely in the future. Hence, it is likely that shallow water coral reefs will be affected more frequently by episodic hypoxic conditions driven by algal blooms. More studies are, however, required to understand the mechanism of coral mortality due to algal blooms, impacts on community composition and the potential for subsequent recovery.

## Introduction

Dissolved oxygen levels in the oceans and coastal waters around the world have been reported to be declining, and coastal ecosystems including coral reefs are affected by this^1,2^. Increased nutrient inputs and warming waters combine to reduce the oxygen levels in global waters^2^. Coral reefs around the world are undergoing severe decline mainly due to issues related to climate such as mass bleaching, ocean acidification and ocean deoxygenation^3–5^. Ocean deoxygenation in particular has increasingly received attention in the context of global coral reef health in recent years^5–7^. In addition to global ocean climate stressors, localised hypoxic events driven by e.g. eutrophication also have the capacity to impact coral communities^1,8,9^. Hypoxia in the marine ecosystem describes a condition of low dissolved oxygen levels that may affect marine organisms by altering behavioural and physiological responses, reducing growth rates and fecundity and, if prolonged or severe, may lead to mortality^5,10^. Ecological processes in a reef ecosystem such as productivity, respiration, reproduction, calcification, bleaching, eutrophication, acidification and space-competition are related to oxygen levels and can be affected by hypoxic conditions^7^. Recent studies underline the severity of hypoxic conditions in the water column and their impact on corals^1,11^.

Temporary hypoxic conditions caused by algal blooms can result in reef-building coral mortality, but are reported rarely^12,13^. Climate change has been linked to increased frequencies of algal blooms in recent years^14^. Frequency of algal blooms, especially of *Noctiluca scintillans*, has increased significantly in the Indian Ocean and in Indian waters during the past few decades^15,16^. Blooms of *N. scintillans* can be of two forms that are red or green tides; red is heterotrophic while green has a photosynthetic symbiont *Pedinomonas noctilucae*^17^. While red tides of *N. scintillans* have been reported to be harmful, green tides have been termed as harmless though they cause low dissolved oxygen levels^18^.

The Gulf of Mannar (GoM) in southeast India is one of the four major coral reef areas of India that provides direct livelihood to thousands of dependent fishermen. GoM has a chain of 21 uninhabited islands between Tuticorin and Rameswaram around which coral reefs mainly occur. Corals of GoM have been disturbed significantly by climate change implications and localised anthropogenic impacts^19,20^. Blooms of *N. scintillans* causing a green tide were witnessed in GoM during September 2019 when thousands of fishes and other organisms were found dead along the shore in the Mandapam region. Previously, algal blooms were not considered a significant threat to corals of GoM, though minimal impact was observed on corals due to a similar bloom in 2008^12^. The present study reports the impact of the September 2019 bloom of *N. scintillans* on corals of GoM.

## Materials and methods

### Preliminary assessment

**Figure 1:**
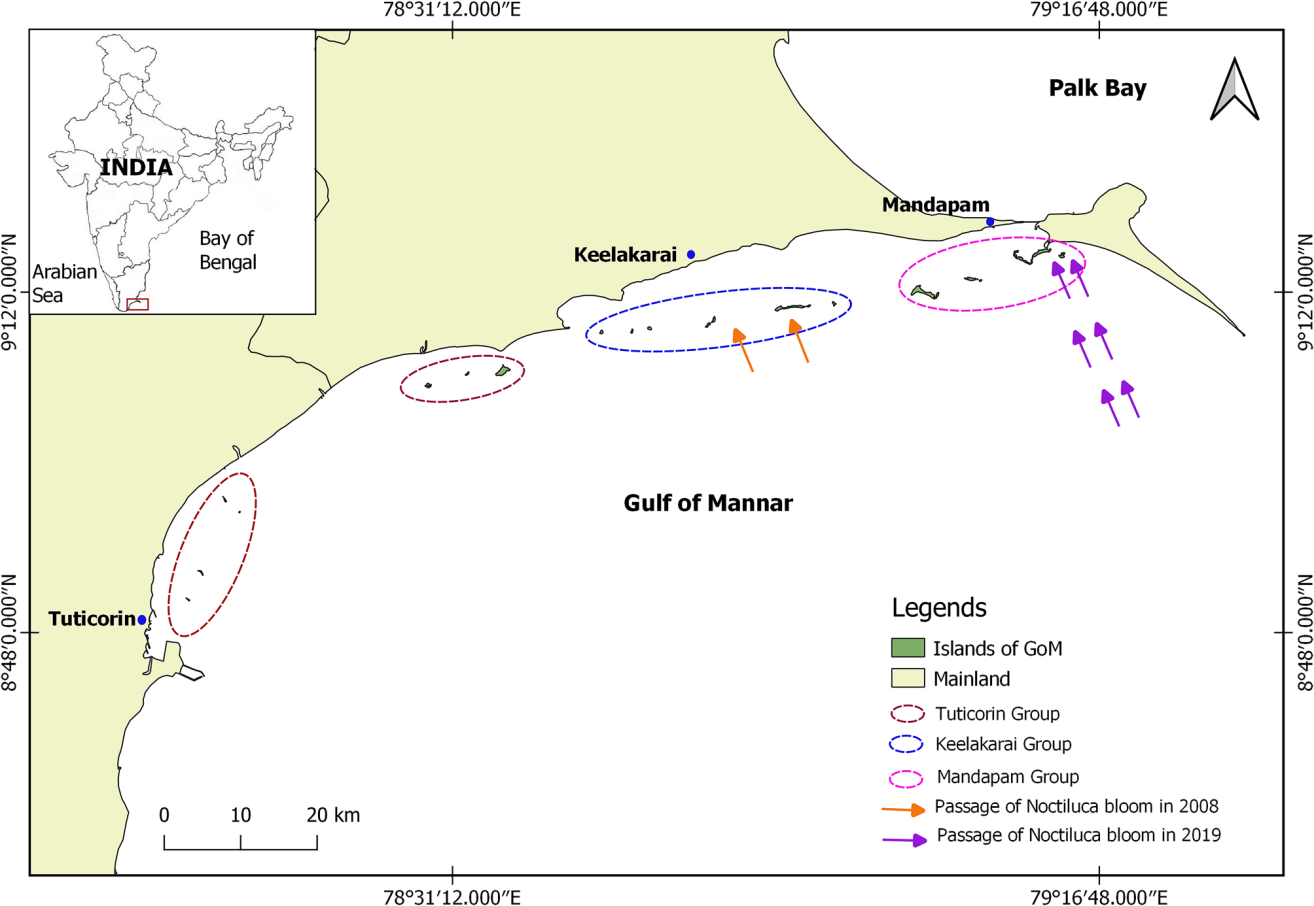
Map showing islands of the Gulf of Mannar and passage of the *Noctiluca scintillans* bloom; Base map was prepared by digitizing the georeferred Toposheet of Survey of India (http://www.surveyofindia.gov.in/) and field data using Open source GIS software (QGIS 3.10.6; https://qgis.org/en/site/forusers/download.html).

The 21 islands of GoM are in three groups with seven islands each, the Mandapam, Keelakarai and Tuticorin groups (Fig. 1). Bloom of *N. scintillans* had affected Keelakarai region of GoM in 2008^12^. On 12th September 2019, a large drifting bloom of *N. scintillans* was found to have killed thousands of fishes along the shore between Kundukal and Vedalai (Approximately 17 km coastal distance) in the Mandapam region (Fig. S1). A rapid underwater visual assessment was done from 14 to 18th of September 2019 to find out the possible impact of the bloom on corals in the Mandapam group of islands, namely Shingle, Krusadai, Pullivasal, Poomarichan, Manoliputti, Manoli and Hare. SCUBA dives were made at arbitrary locations around these islands to do visual inspection of corals to document the possible bleaching and mortality. This preliminary assessment confirmed impact of the bloom at two islands, Shingle and Krusadai. The area of impact was delineated and measured with GPS tracking and the data were transferred into QGIS (Quantum GIS) open source software.

### Detailed assessment

Twenty meter (20X2m) belt transects were laid to assess the prevalence of affected coral colonies in the affected areas of Shingle and Krusadai Islands on 17th and 27th September and 04th October 2019. Ten belt transects (20X2m) were laid at the impacted areas in Shingle Island and five at Krusadai Island with a minimum distance of 10 m between transects. Colony counts were made to assess the prevalence of affected colonies within the 40 m^2^ area of each transect. On each sampling visit, the same number of transects were laid at each island, but transect placement was arbitrary each time within the affected area. Samples of *N. scintillans* cells were collected from the surface water by towing a plankton net number 30 of mesh size 60 µm for half an hour and the concentration density was measured using a Sedwick Rafter counting chamber.

Water samples were collected from the affected areas each time and dissolved oxygen content was measured using Winkler’s titration method following Strickland and Parsons^21^. The depth of the reef was very shallow (< 3) and coral growth was up to 1 m above the bottom. To measure the dissolved oxygen content, triplicate water samples were collected in 125 ml BOD bottles with glass stoppers just above the coral colonies (1–2 m depth) by diving, between 8 and 9 AM in the morning. Samples were immediately brought to the boat for immediate fixing with 1 ml of manganous chloride and 1 ml of alkaline iodide, and shaken vigourously to stimulate precipitation. 1 ml of concentrated sulfuric acid was then added to dissolve the precipitate before the titration, ensuring no air bubbles were trapped inside. Fixed samples were kept inside a dark box until titration. Titration of the fixed samples was done with sodium thiosulfate and starch solution as an indicator, within two hours of collection. Study islands are very close to the shore and hence the analysis could be done within two hours in the field centre.

Water samples were collected at the same depth to measure temperature, salinity, pH and TDS (Total Dissolved Solids) in a sterile plastic container. Water temperature was measured using a digital thermometer and salinity was measure using a handheld refractometer. pH and TDS were measured in the field using a handheld water proof tester Hanna HI98129. All these parameters were measured immediately on the boat.

## Results and discussion

**Figure 2:**
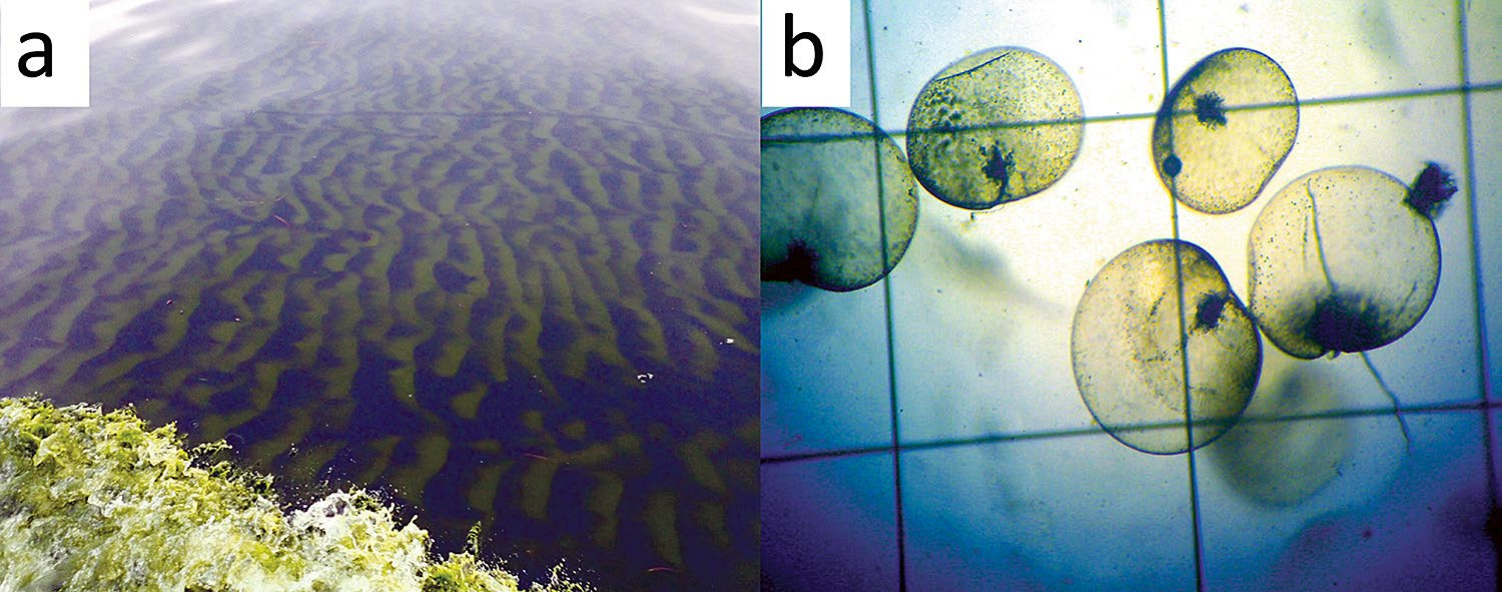
(**a**) Green tide of *Noctiluca scintillans* in the Gulf of Mannar; (**b**) image of *N.scintillans* cells; size of the grid is 1 mm^2^ (*N. scintillans* exhibits bioluminescence when disturbed).

**Figure 3:**
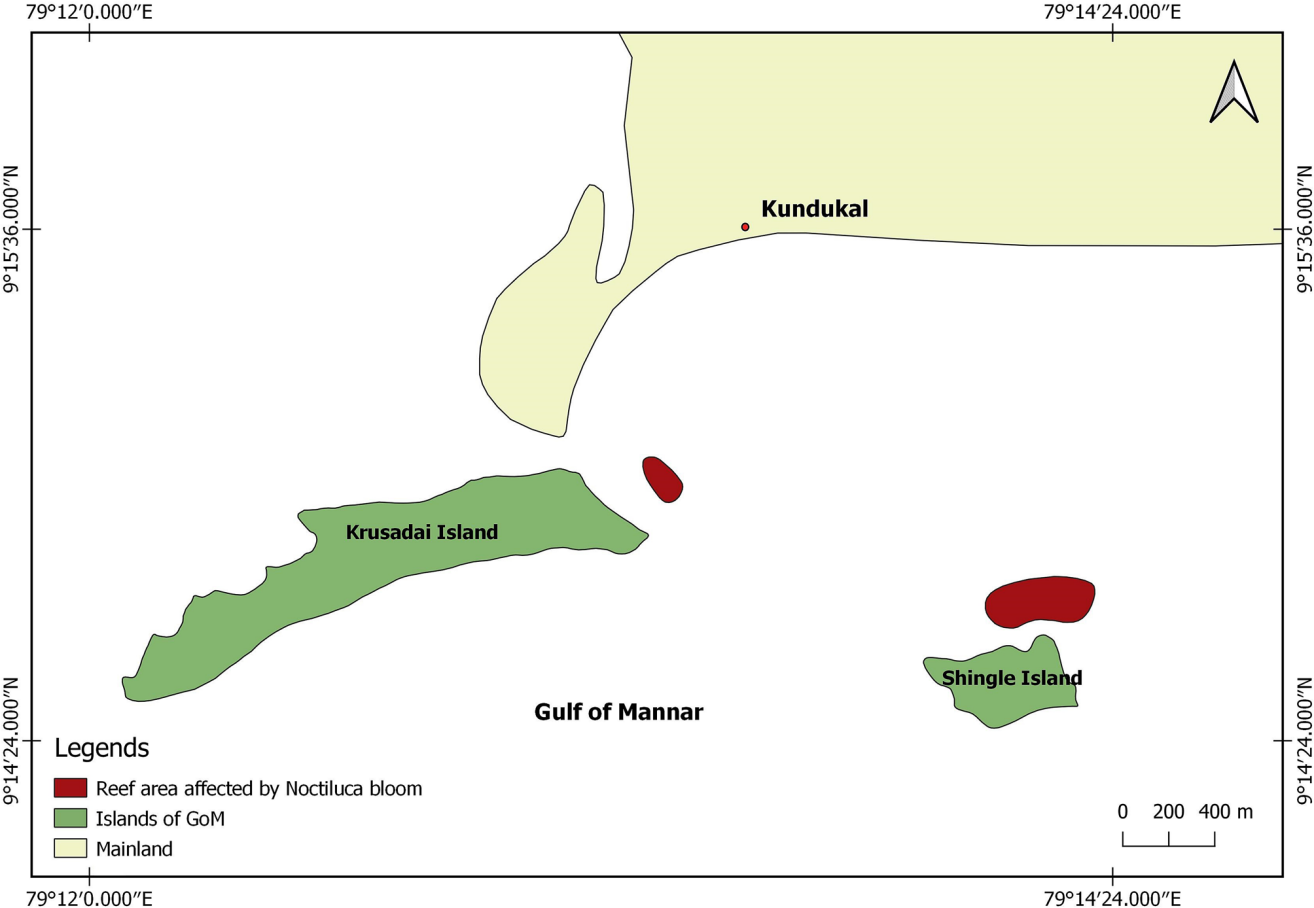
Map showing the affected islands in the Mandapam group shown in Fig. 1. Base map was prepared by digitizing the georeferred Toposheet of Survey of India (http://www.surveyofindia.gov.in/) and field data using Open source GIS software (QGIS 3.10.6; https://qgis.org/en/site/forusers/download.html).

Though the time and place of the origin of this bloom is unknown, the presumable causes of it were high temperatures, abundant nutrients, low tidal amplitude, and little current. According to fishermen, these bioluminescent blooms were first seen about 15 nautical miles offshore of the Mandapam coast between India and Sri Lanka on 6th September, and subsequently moved towards the shore (Fig. 2). Bloom of *N. scintillans* in 2008 was reported to affect all the marine organisms including corals in GoM^12^. On 14th September, our preliminary assessment revealed that corals in Shingle and Krusadai islands were possibly affected by the bloom. A great multitude of *N. scintillans* cells were found settled on corals and other benthic organisms in the affected areas. A greenish settlement was observable on live coral colonies and other benthic organisms including macro algae, coralline algae and sponges etc.(Fig. S2). Settling of *N. scintillans* on benthic organisms has been reported to cause significant damage to the reef organisms through asphyxiation^12^. At Shingle Island, the area of significant impact was about 8.1 hectares on the shoreward side of the Island (79°14′14.38″E, 9°14′44.23″N) at depths between 1 and 3 m (Fig. 3). At Krusadai Island, an area of 2.1 hectares in the shoreward side was found affected by the bloom (79°13′20.78″E, 9°15′00.88″N) at depths between 1 and 2 m. The rest of the reef areas in both of these islands were healthy without any impact. The settled cells of *N. scintillans* were found to be washed ashore during subsequent surveys. In addition to dead fishes, a multitude of benthic communities such as crustaceans, mollusks and echinoderms were also found dead on the bottom in the impacted areas. Surveys between 15 and 18th September 2019 confirmed that corals in other islands (Pullivasal, Poomarichan, Manoliputti, Manoli and Hare) were in good health, and without any noticeable impact due to the bloom. Shingle and Krusadai islands occur closest to the mainland, and the concentrated bloom appeared to get trapped by currents between the mainland shore and islands.

On 14th September, coral mortality was not observed in the affected areas though the colonies were observed to be disturbed by the settling *N. scintillans* cells. Low dissolved oxygen levels have been reported to be the primary cause of benthic mortality during algal blooms^22^. Dissolved oxygen levels were 1.48 mg l^−1^ at Shingle Island and 2.02 mg l^−1^ at Krusadai Island in the affected areas. This compares to ‘normal’ levels for coral reefs of 5–8 mg l^−1^, and Haas et al.^11^ found that dissolved oxygen content less than 4 mg l^−1^ is detrimental to acroporid corals. Moreover, branching coral forms have been reported to be more susceptible to hypoxic episodes than spherical or massive forms^5^. Corals are routinely exposed to fluctuations in oxygen levels at the tissue level due to photosynthesis and respiration processes of endosymbionts^7^, but are negatively impacted when (sub-) lethal thresholds of hypoxia exposure are exceeded^1,5,11^. Lethal hypoxia thresholds appear to differ considerably between coral species, ranging between 0.5 and 4 mg O2 l^−1^^1,5,11^, while sub-lethal hypoxia thresholds for corals are almost entirely unknown^5^.

Seawater temperature can significantly impact dissolved oxygen levels^23,24^. Water temperature was 29.9 and 29.8º C (Table 1) at Shingle and Krusadai islands respectively and these levels are marginally higher than the normal levels for this particular time of the year. Apart from the summer months (April to June), temperature levels in GoM do not go higher than 29º C^20^. The concentration of *N. scintillans* was 43.4 × 10^5^ and 27.3 × 10^5^ cells l^−1^ at Shingle and Krusadai Islands respectively; pH and TDS were also high in the affected area (Table 1). Dissolved oxygen levels in other sites of these two islands and in other five islands were higher than 5 mg l^−1^.

**Table 1:** Environmental characterization at the affected sites in Shingle and Krusadai Islands.

Date	*Noctiluca scintillans* density (cells l^−1^)	Dissolved Oxygen content (mg l^−1^)	Temperature (°C)	Salinity (PPT)	pH	TDS (Total Dissolved Solids) (g/l)
**Shingle Island**						
14th September 2019	43.4 × 10^5^	1.48	29.9	33.2	7.08	51.44
17th September 2019	1.63 × 10^3^	3.78	28.4	34.2	8.05	46.96
27th September 2019	Nil	6.02	29.5	34.8	8.24	28.62
4th October 2019	Nil	7.13	28.8	35	7.13	27.54
**Krusadai Island**						
14th September 2019	27.3 × 10^5^	2.02	29.8	33.4	7.12	49.5
17th September 2019	0.88 × 10^3^	3.39	28.5	34.2	8.1	49.34
27th September 2019	Nil	5.73	29.6	34.5	8.32	19.14
4th October 2019	Nil	7.24	28.6	35.1	7.24	30.89

**Figure 4:**
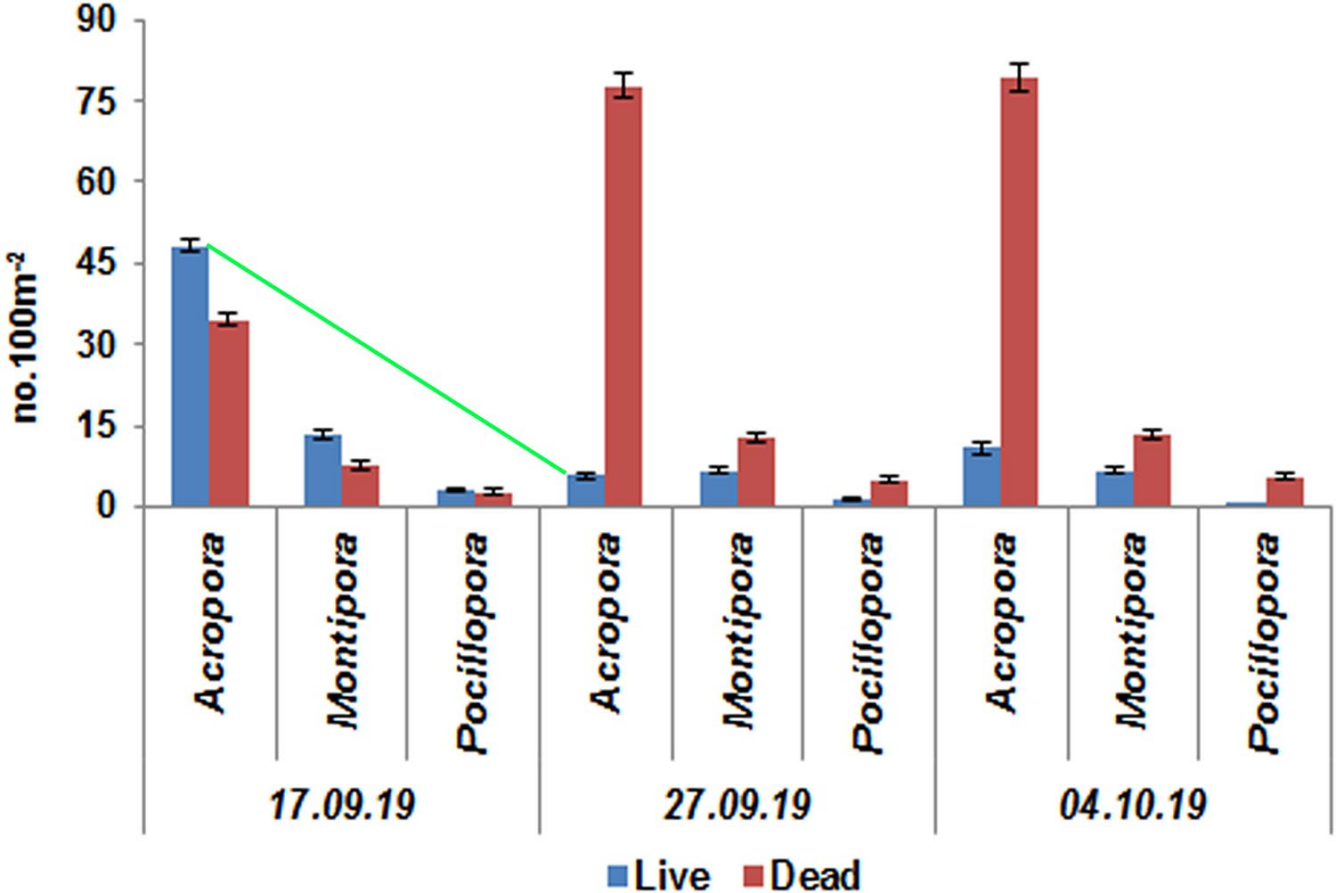
Density of live and dead colonies of affected coral genera (*Acropora, Montipora* and *Pocillopora*) in Shingle Island, by date; the green line indicates the drastic decline of *Acropora* density between 17.09.2019 and 27.09.2019.

**Figure 5:**
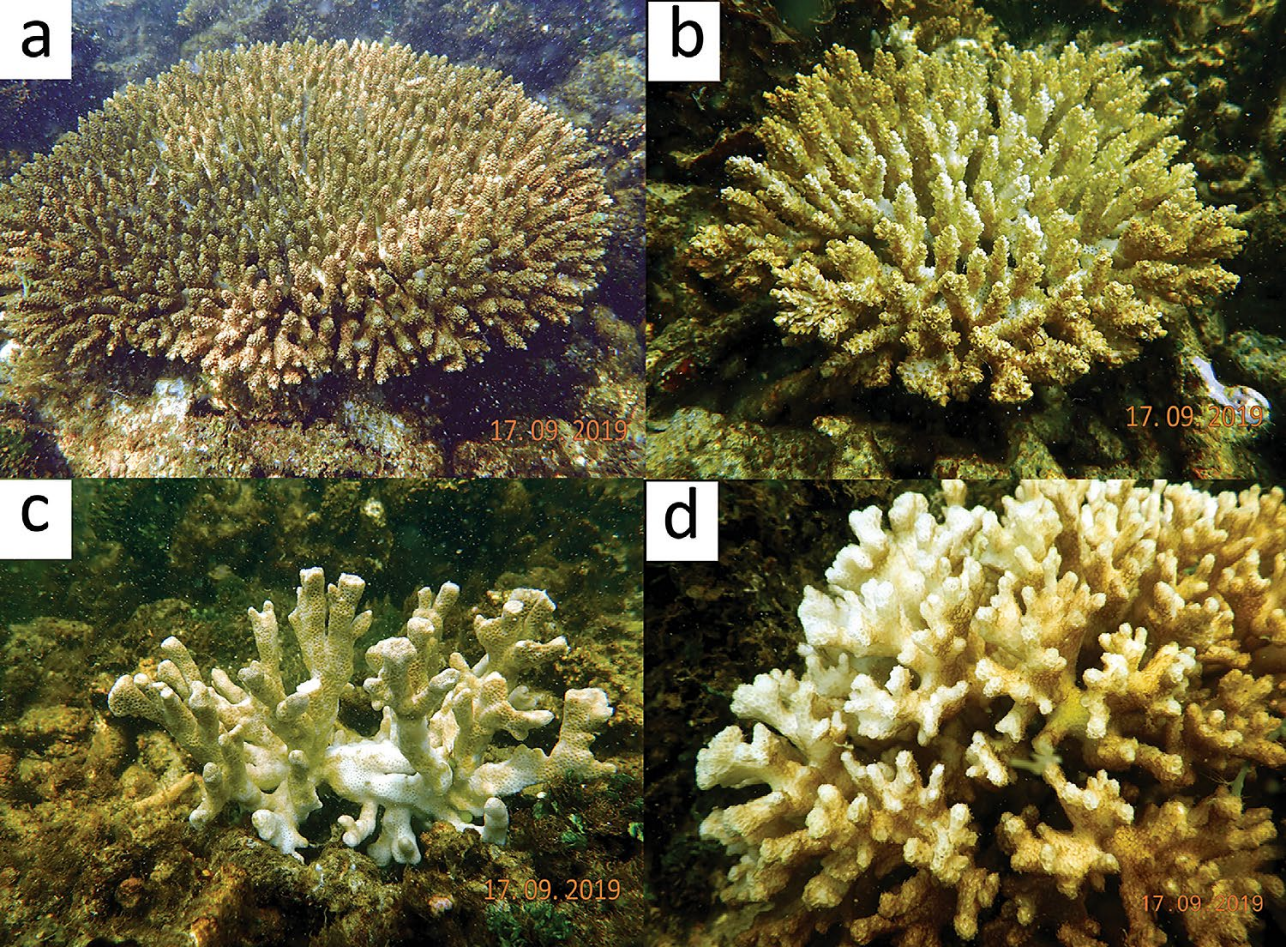
Rapid mortality of corals presumably due to low oxygen levels caused by *Noctiluca scintillans*; (**a**, **b**) *Acropora*; (**c**) *Montipora*; (**d**) *Pocillopora.*

During the next assessment on 17th of September 2019, severe coral mortality was observed at the affected sites. At Shingle Island, overall coral colony density was 134.25 (SE ± 3.28) no.100 m^−2^ (n = 537) within ten 20 m belt transects which is dominated by *Acropora* (64%) followed by *Montipora* (15%). Out of total 537 colonies, 33.52% (n = 180) were found dead (Fig. 4), which include 34.5 (SE ± 1.05) no.100 m^−2^ (n = 138) of *Acropora*, 7.75 (SE ± 0.75) no.100 m^−2^ (n = 31) of *Montipora* and 2.75 (SE ± 0.35) no.100 m^−2^ (n = 11) of *Pocillopora*. The death of coral colonies was so rapid that the coral tissue was intact on the colony surface and still had its natural colour (Fig. 5). When wafted with water by hand or with scuba air, the tissue peeled off exposing the skeleton (Supplementary video). Other observed genera such as *Dipsastraea, Favites, Porites, Hydnophora, Goniastrea, Echinopora, Turbinaria, Platygyra, Goniopora* and *Symphyllia* in the same site were all alive (Fig. S3), though with excess mucus production. This may be explained by differential lethal thresholds for oxygen levels at species and growth form levels^5,19^. At Krusadai Island, the overall coral density on 17th September was 66 (SE ± 2.54) no.100 m^−2^ (n = 132), dominated by *Acropora*. Among the counted colonies, 6 (SE ± 1.03) no.100 m^−2^ of *Acropora* were found recently dead while mortality was not observed in other available genera such as *Montipora, Pocillopora, Dipsastraea, Favites, Porites* and *Turbinaria*. Dissolved oxygen levels had increased to 3.78 mg l^−1^ at Shingle Island and to 4.02 mg l^−1^ at Krusadai Island at the affected sites and the water had started to become clear. The concentration of *N. scintillans* had reduced to 1.63 × 10^3^ cells l^−1^ and 0.88 × 10^3^ cells l^−1^ at Shingle and Krusadai Islands, respectively (Table 1).

Assessment on 27th September 2019 at the impacted area in Shingle Island, showed that the overall density of coral colonies within ten 20 m transects was 135.75 (SE ± 2.82) no.100 m^−2^ (n = 543) and of them 70.35% (n = 382) of colonies belonging to *Acropora*, *Montipora* and *Pocillopora* were found dead revealing that the impact of algal bloom was more severe than expected (Fig. 4). It was almost two weeks since the corals had died and hence secondary algae had started colonizing the dead colonies. On the same day at the impacted area of Krusadai Island, overall coral density within five belt transects was 65.5 (SE ± 1.83) no.100 m^−2^ (n = 131), of which 9.09% (n = 12) of colonies belonging to *Acropora* were found dead. By 27th September, dissolved oxygen levels had increased to 6.02 and 5.73 mg l^−1^ respectively at the affected areas of Shingle and Krusadai islands (Table 1). *N. scintillans* cells were absent in all the sites indicating the end of bloom. On 04th October 2019, the overall coral colony density within 20 m belt transects was 138 (SE ± 2.08) no.100 m^−2^ (n = 552) and of them 71.23% (n = 393) colonies belonging to *Acropora*, *Montipora* and *Pocillopora* were found dead at the area of impact in Shingle Island (Fig. 4). No further mortality was witnessed in the affected area of Krusadai Island. Secondary algae have completely overgrown the dead coral colonies making the reef look green (Fig. S4). Dissolved oxygen levels were reasonably high at 7.13 and 7.24 mg l^−1^ respectively at Shingle and Krusadai Islands during this time (Table 1).

Coral mortality due to algal bloom and consequent hypoxia has rarely been reported^12,13,25^. The present study reports that the impact of blooms can be severe on corals. Different coral species respond differently to low oxygen levels according to their respiration and photosynthesis^5,26^. Thus, low oxygen levels can orchestrate the coral mortality by affecting coral’s productivity and respiration^7^. Further, fast growing corals such as *Acropora* and *Pocillopora* have been reported to be more susceptible to low oxygen levels^11,13,27^. Fast growing coral species have faster metabolism rates^28^ and hence metabolic oxygen requirements are higher^11,29^. Thus, the mortality of fast growing species in the present study was presumably due to the low oxygen levels induced by *N.scintillans* bloom.

Bleaching episodes in 2010 and 2016 had also caused significant mortality to these fast growing species in GoM^19,20^. Corals in GoM start to bleach when water temperature exceeds 30º C and the temperature levels during this bloom period ranged between 28.4 and 29.9º C. Though bleaching was not observed, heat stress might also have played its role in coral mortality along with low oxygen levels as the temperature level almost reached 30º C. Similar temperature levels were reported during the bloom of *N. scintillans* in 2008 in GoM^12^.

Corals in Gulf of Mannnar are still recovering from the 2016 bleaching episode^20^ and hence the present decline is significant. Phase shifts on coral reefs are predominantly associated with shifts from hard coral-dominated communities to macroalgae-dominated ones^30^. Space competition between corals and other organisms such as algae and sponges has been reported to negatively impact the corals of GoM after the 2016 bleaching event^20,31^. At present, secondary algae have completely occupied the dead coral colonies, which will affect the coral recovery by hindering the attachment of new coral recruits during the next spawning season^32^. Recent studies suggest hypoxia increases coral susceptibility to bleaching^27^, and may increase disease prevalence and algal proliferation^7^. Thus algal blooms add to the existing array of threats to corals of GoM that needs to be understood more with further focused research.

On account of the problems related to climate change, there has been a steady and severe decline of coral reefs in the past two decades. Bleaching and diseases have been reported to cause mass coral mortalities within a very short time. The observations of the present study alert us to possible mass mortality due to short-term hypoxic condition caused by algal blooms. Algal blooms and hypoxic conditions are predicted to occur more frequently in the future due to climate change^14^. Hence, it is likely that shallow water coral reefs will be affected more frequently by temporary low oxygen levels caused by algal blooms. More studies are, however, required to understand the mechanism of coral mortality due to algal blooms, impacts on community composition and the potential for subsequent recovery.

## Supplementary information

Supplementary Figures.

Supplementary Video.
