# Supplementary material for: Low oxygen levels caused by Noctiluca scintillans bloom kills corals in Gulf of Mannar, India
Source: Sci Rep. 2020 Dec 17;10:22133. doi: 10.1038/s41598-020-79152-x (PMC7746711; doi:10.1038/s41598-020-79152-x)
Supplement: Supplementary file 1 — Supplementary Figures. [file 41598_2020_79152_MOESM1_ESM.pdf]

Low oxygen levels caused by *Noctiluca scintillans* bloom kills corals in Gulf of Mannar, India

*K. Diraviya Raj[1], G. Mathews[1], David O. Obura[2], R. L. Laju[1], M. Selva Bharath[1], P. Dinesh Kumar[1], A. Arasamuthu[1], T. K. Ashok Kumar[3] and J. K. Patterson Edward[1]

[1] Suganthi Devadason Marine Research Institute, 44-Beach Road, Tuticorin 628001, Tamil Nadu, India

[2] CORDIO East Africa, P.O.BOX 10135, Mombasa, 80101, Kenya

[3]Gulf of Mannar Marine National Park, Ramanathapuram 623 503, India

*Corresponding author: diraviyam_raj@yahoo.co.in

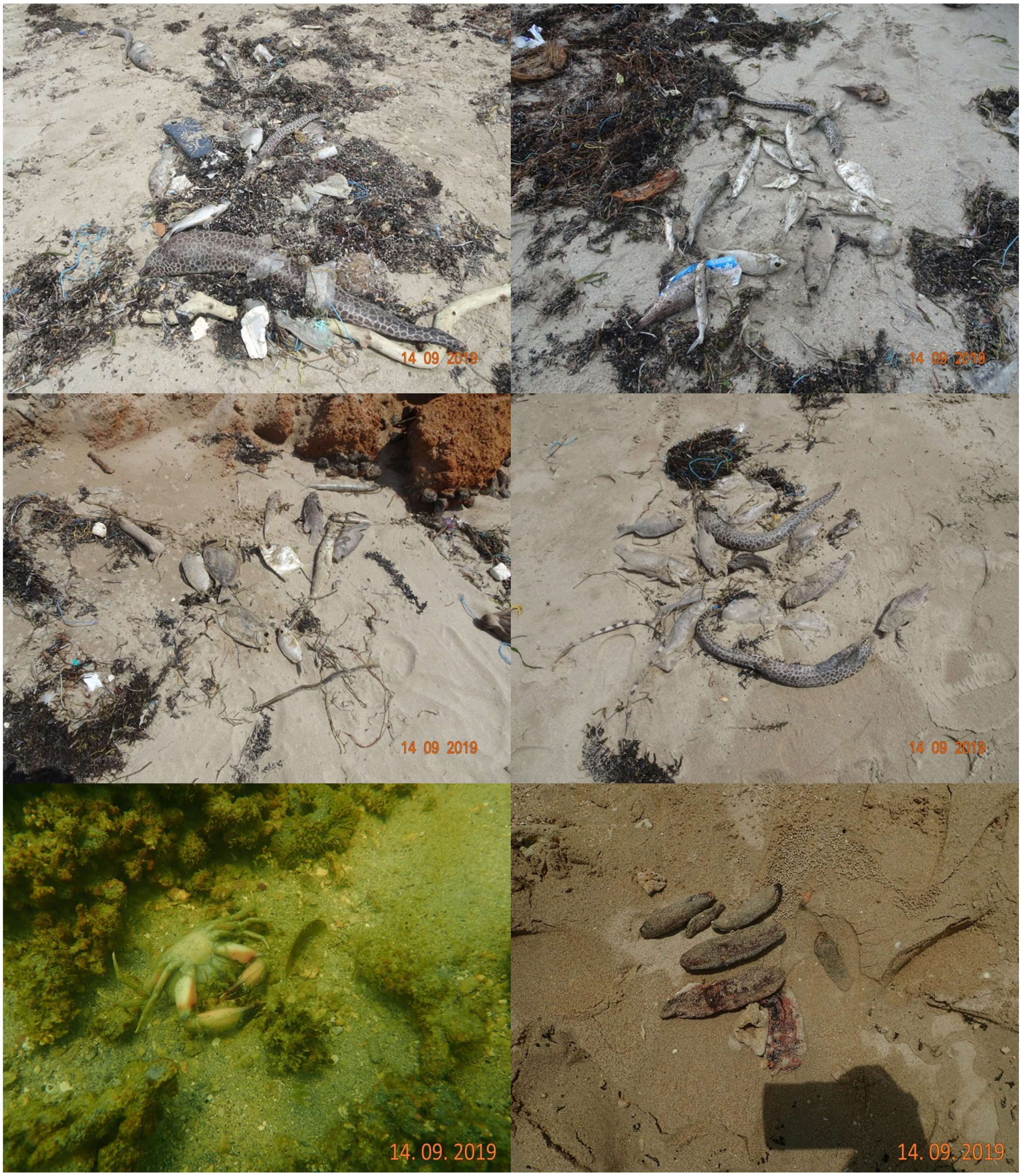

Fig. S1. Mortality of marine organisms caused by the bloom of *Noctiluca scintillans* along the coast between Kundukal and Vedalai

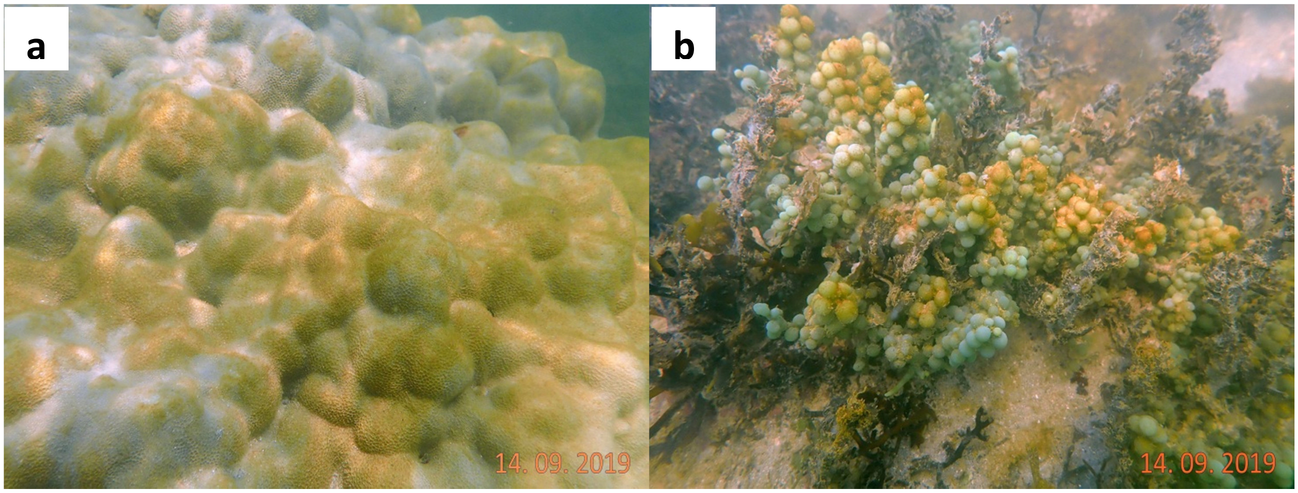

Fig. S2. Settlement of *Noctiluca scintillans* cells on benthic organisms; a, settlement on a coral colony (*Porites* sp.); b, settlement on macroalga (*Caulerpa* sp.)

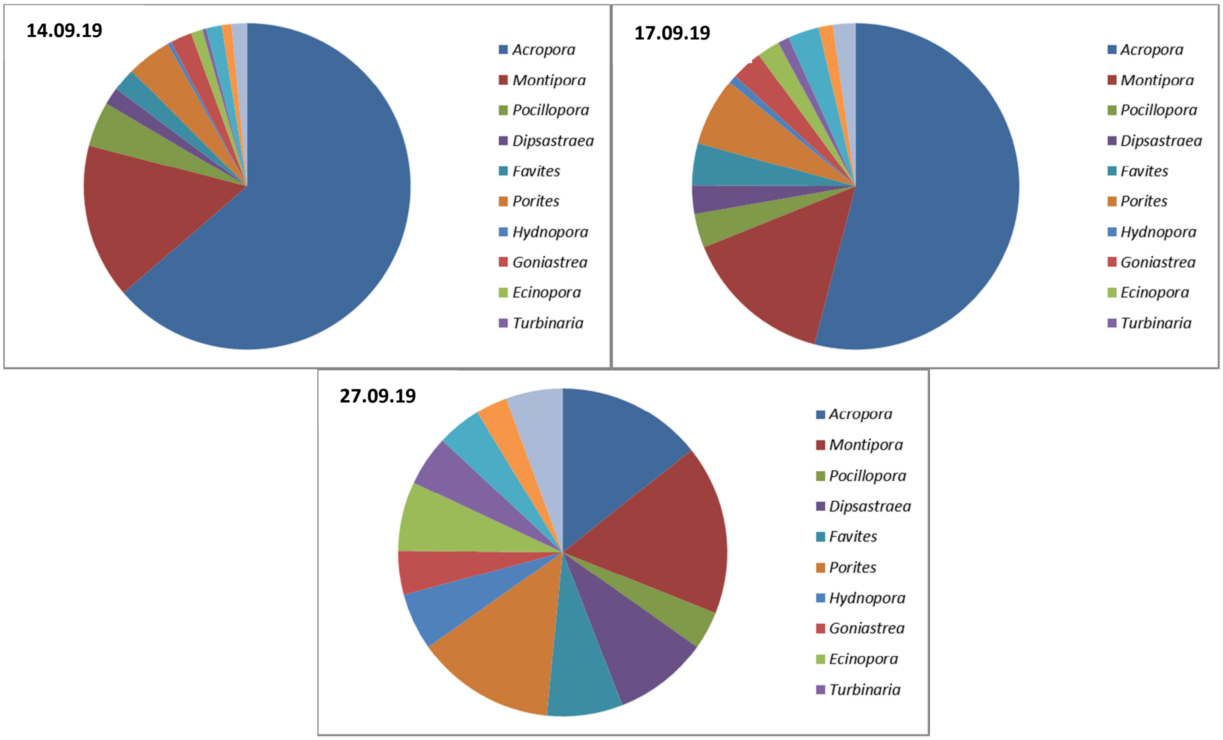

Fig. S3. Change of coral community due to the mortality caused by algal bloom in Shingle Island, Gulf of Mannar

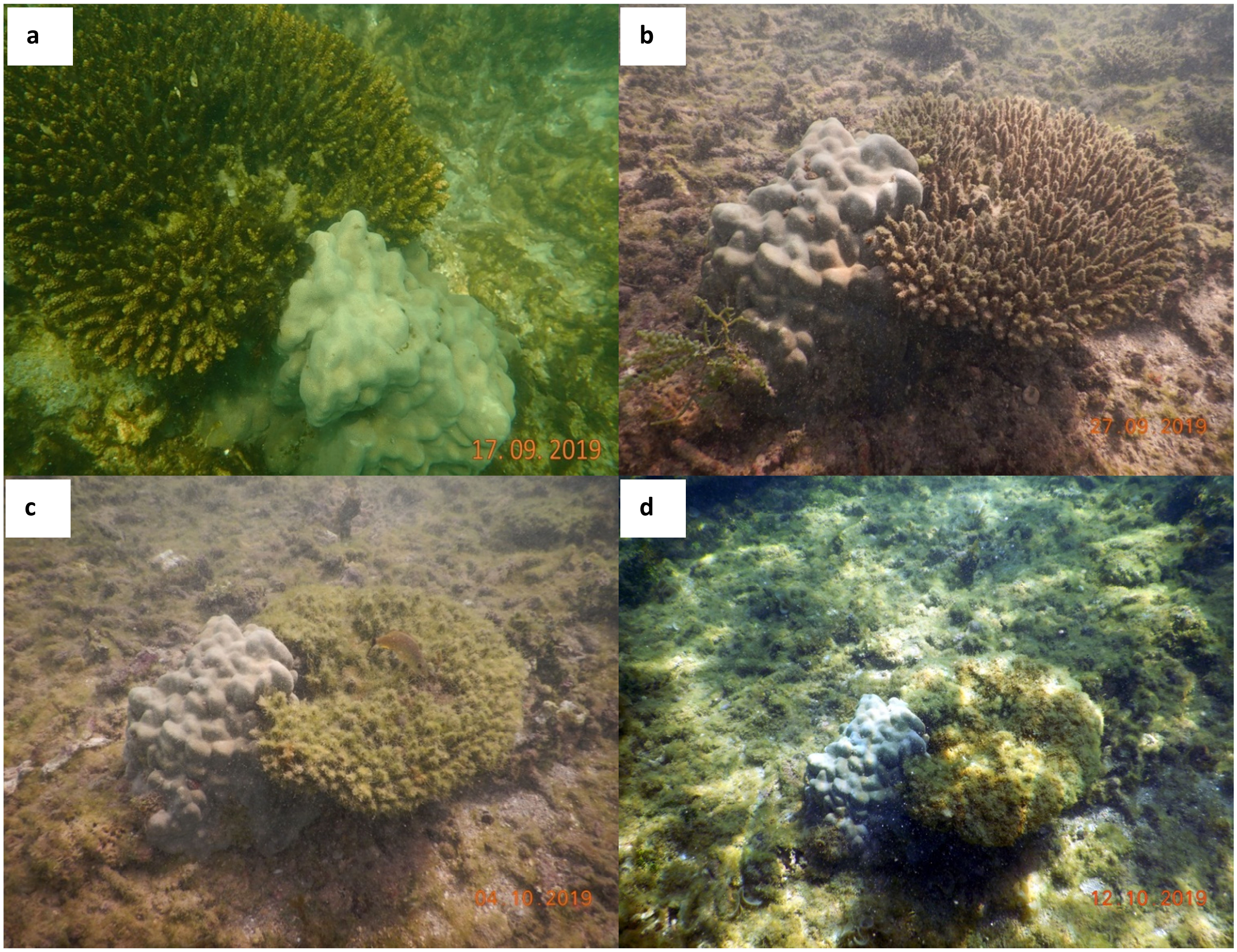

Fig. S4. Fate of an *Acropora* colony affected by *Noctiluca scintillans* bloom; a, photo taken on 17.09.19; b, photo taken on 27.09.19; c, photo taken on 04.10.19; d, photo taken on 12.10.19
